# Dibromidobis(pyridine-3-carbonitrile-κ*N* ^1^)mercury(II)

**DOI:** 10.1107/S1600536811013274

**Published:** 2011-04-16

**Authors:** Reza Ghiasi

**Affiliations:** a Department of Chemistry, Basic Science Faculty, East Tehran Branch, Islamic Azad University, Qiam Dasht, Tehran, Iran

## Abstract

In the crystal structure of the title compound, [HgBr_2_(C_6_H_4_N_2_)_2_], the Hg atom is four coordinated by two pyridine N atoms and two Br^−^ anions in a considerably distorted tetrahedral environment. π–π inter­actions between adjacent pyridine rings [centroid–centroid distance of 3.648 (3) Å] stabilize the crystal structure.

## Related literature

For related structures, see: Ghiasi (2011 ▶); Steffen & Palenik (1977 ▶); Li *et al.* (2004 ▶).
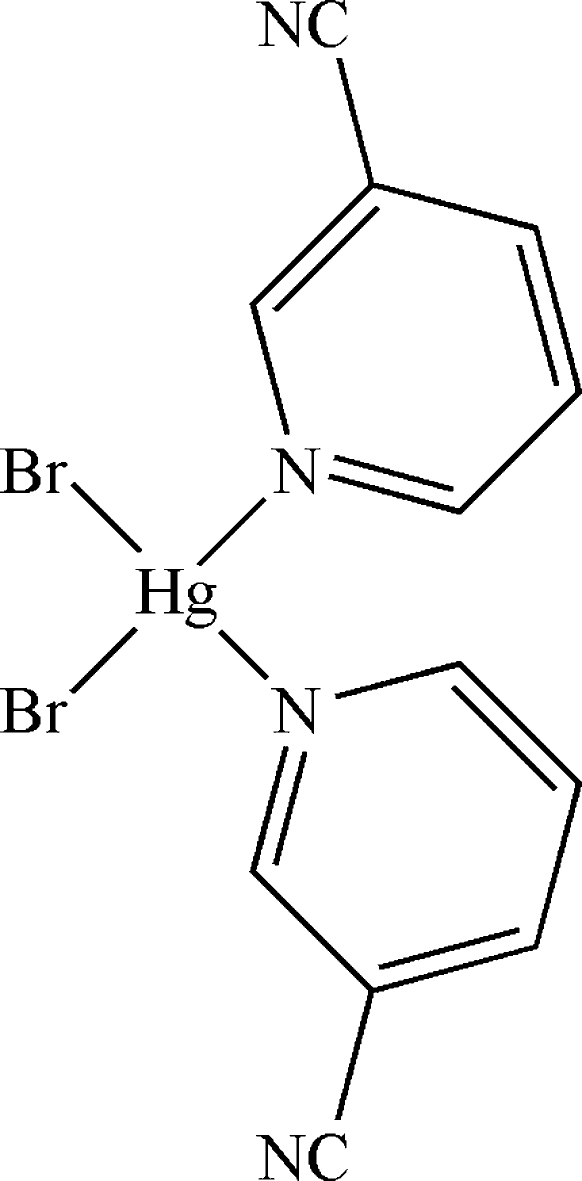


## Experimental

### 

#### Crystal data

[HgBr_2_(C_6_H_4_N_2_)_2_]
*M* *_r_* = 568.61
Triclinic, 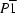

*a* = 8.5823 (6) Å
*b* = 9.4069 (6) Å
*c* = 9.8562 (7) Å
α = 81.935 (5)°
β = 71.435 (6)°
γ = 80.508 (6)°
*V* = 740.70 (9) Å^3^
*Z* = 2
Mo *K*α radiation
μ = 15.78 mm^−1^
*T* = 120 K
0.45 × 0.22 × 0.20 mm

#### Data collection

Bruker SMART CCD diffractometer
Absorption correction: multi-scan (*SADABS*; Bruker, 1998 ▶) *T*_min_ = 0.033, *T*_max_ = 0.052
8486 measured reflections
3967 independent reflections
3751 reflections with *I* > 2σ(*I*)
*R*_int_ = 0.043

#### Refinement

*R*[*F* ^2^ > 2σ(*F* ^2^)] = 0.030
*wR*(*F* ^2^) = 0.084
*S* = 1.18
3967 reflections
172 parameters
H-atom parameters constrained
Δρ_max_ = 1.13 e Å^−3^
Δρ_min_ = −2.48 e Å^−3^

### 

Data collection: *SMART* (Bruker, 1998 ▶); cell refinement: *SAINT* (Bruker, 1998 ▶); data reduction: *SAINT*; program(s) used to solve structure: *SHELXTL* (Sheldrick, 2008 ▶); program(s) used to refine structure: *SHELXTL*; molecular graphics: *ORTEP-3 for Windows* (Farrugia, 1997 ▶); software used to prepare material for publication: *WinGX* (Farrugia, 1999 ▶).

## Supplementary Material

Crystal structure: contains datablocks global, I. DOI: 10.1107/S1600536811013274/bt5511sup1.cif

Structure factors: contains datablocks I. DOI: 10.1107/S1600536811013274/bt5511Isup2.hkl

Additional supplementary materials:  crystallographic information; 3D view; checkCIF report

## Figures and Tables

**Fig. 1.**
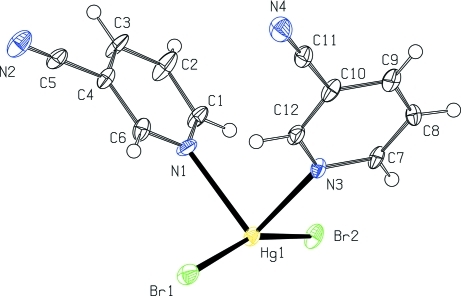
The molecular structure of the title molecule, with the atom-numbering scheme. Displacement ellipsoids are drawn at the 50% probability level.

**Fig. 2.**
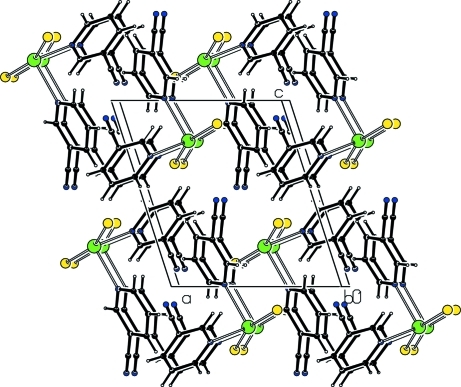
Unit-cell packing diagram for (I).

## supplementary crystallographic information

### Comment

Recently, the crystal satructure of dibromozinc(II)-di-3-pyridine-carbonitrile have been reported, (Ghiasi, 2011). On the other hand there are several complexes, with formula, [*MX*_2_*L*_2_], such as [ZnCl_2_(4-cypy)_2_], (Steffen & Palenik, 1977), [CuBr_2_(3-Cypy)_2_], (Li *et al.* 2004), [where py is pyridine, 4-cypy is 4-cyanopyridine and 3-cypy is 3-cyanopyridine] have been synthesized and characterized by single-crystal X-ray diffraction methods. The molecular structure of the title compound is shown in Fig. 1. The Hg^II^ atom is four-coordinated in a slightly distorted tetrahedral configuration by two N atoms from two pyridine rings and two Br^-^ anions. The Hg—Br and Hg—N bond distances and angles (Table 1) are within normal ranges.  π-π interactions between adjacent pyridine rings [centroid···centroid distance of 3.648 (3) Å, symmetry code: –*x*,1-y,1-*z*] stabilize the packing of the crystal structure.

### Experimental

Mercury(II) bromide (0.72 gr, 2 mmol) was disolved in methanol (12 ml) and the solution was mixed with a methanolic solution (10 ml) of 3-pyridinecarbonitrile (0.42 g, 4 mmol). This solution was left to evaporate slowly at room temperature. After one week, colorless prismatic crystals of the title compound were isolated (yield 0.64 g, 56.0%, m.p. < 570 K).

### Refinement

All H atoms were positioned geometrically, with C—H=0.96Å aromatics hydrogen atoms and constrained to ride on their parent atoms, with *U*_iso_(H)=1.2*U*_eq_.

### Crystal data

**Table:**

[HgBr_2_(C_6_H_4_N_2_)_2_]	*Z* = 2
*M**_r_* = 568.61	*F*(000) = 516
Triclinic, *P*1	*D*_x_ = 2.549 Mg m^−^^3^
Hall symbol: -P 1	Mo *K*α radiation, λ = 0.71073 Å
*a* = 8.5823 (6) Å	Cell parameters from 8405 reflections
*b* = 9.4069 (6) Å	θ = 2.2–29.2°
*c* = 9.8562 (7) Å	µ = 15.78 mm^−^^1^
α = 81.935 (5)°	*T* = 120 K
β = 71.435 (6)°	Prism, colorless
γ = 80.508 (6)°	0.45 × 0.22 × 0.2 mm
*V* = 740.70 (9) Å^3^	

### Data collection

**Table:**

Bruker SMART CCD diffractometer	3751 reflections with *I* > 2σ(*I*)
graphite	*R*_int_ = 0.043
phi and ω scans	θ_max_ = 29.2°, θ_min_ = 2.2°
Absorption correction: multi-scan (*SADABS*; Bruker, 1998)	*h* = −11→11
*T*_min_ = 0.033, *T*_max_ = 0.052	*k* = −12→12
8486 measured reflections	*l* = −13→13
3967 independent reflections	

### Refinement

**Table:**

Refinement on *F*^2^	Primary atom site location: structure-invariant direct methods
Least-squares matrix: full	Secondary atom site location: difference Fourier map
*R*[*F*^2^ > 2σ(*F*^2^)] = 0.030	Hydrogen site location: inferred from neighbouring sites
*wR*(*F*^2^) = 0.084	H-atom parameters constrained
*S* = 1.18	*w* = 1/[σ^2^(*F*_o_^2^) + (0.0481*P*)^2^ + 0.8751*P*] where *P* = (*F*_o_^2^ + 2*F*_c_^2^)/3
3967 reflections	(Δ/σ)_max_ = 0.013
172 parameters	Δρ_max_ = 1.13 e Å^−^^3^
0 restraints	Δρ_min_ = −2.48 e Å^−^^3^

### Special details

**Table:**

Geometry. All e.s.d.'s (except the e.s.d. in the dihedral angle between two l.s. planes) are estimated using the full covariance matrix. The cell e.s.d.'s are taken into account individually in the estimation of e.s.d.'s in distances, angles and torsion angles; correlations between e.s.d.'s in cell parameters are only used when they are defined by crystal symmetry. An approximate (isotropic) treatment of cell e.s.d.'s is used for estimating e.s.d.'s involving l.s. planes.
Refinement. Refinement of *F*^2^ against ALL reflections. The weighted *R*-factor *wR* and goodness of fit *S* are based on *F*^2^, conventional *R*-factors *R* are based on *F*, with *F* set to zero for negative *F*^2^. The threshold expression of *F*^2^ > σ(*F*^2^) is used only for calculating *R*-factors(gt) *etc*. and is not relevant to the choice of reflections for refinement. *R*-factors based on *F*^2^ are statistically about twice as large as those based on *F*, and *R*- factors based on ALL data will be even larger.

### Fractional atomic coordinates and isotropic or equivalent isotropic displacement parameters (Å^2^)

**Table:**

	*x*	*y*	*z*	*U*_iso_*/*U*_eq_	
C1	0.0093 (6)	0.7134 (5)	0.4103 (5)	0.0225 (9)	
H1	0.0187	0.784	0.4635	0.027*	
C2	−0.1379 (6)	0.6544 (5)	0.4499 (6)	0.0239 (9)	
H2	−0.2248	0.6851	0.5286	0.029*	
C3	−0.1559 (7)	0.5491 (5)	0.3719 (5)	0.0241 (9)	
H3	−0.2534	0.5073	0.3971	0.029*	
C4	−0.0218 (6)	0.5089 (5)	0.2548 (5)	0.0224 (9)	
C5	−0.0290 (6)	0.4010 (5)	0.1668 (6)	0.0241 (9)	
C6	0.1248 (7)	0.5712 (5)	0.2222 (5)	0.0227 (9)	
H6	0.2146	0.5409	0.1454	0.027*	
C7	0.2284 (7)	1.0208 (6)	−0.0244 (6)	0.0277 (10)	
H7	0.1906	1.0754	0.0541	0.033*	
C8	0.1848 (9)	1.0747 (6)	−0.1468 (6)	0.0355 (13)	
H8	0.1204	1.1641	−0.1508	0.043*	
C9	0.2387 (8)	0.9933 (6)	−0.2636 (6)	0.0308 (11)	
H9	0.2099	1.0259	−0.347	0.037*	
C10	0.3368 (6)	0.8616 (5)	−0.2530 (5)	0.0221 (9)	
C11	0.3959 (7)	0.7725 (6)	−0.3706 (6)	0.0265 (10)	
C12	0.3758 (7)	0.8154 (5)	−0.1251 (5)	0.0237 (9)	
H12	0.4413	0.7271	−0.118	0.028*	
Br1	0.59621 (7)	0.60216 (6)	0.12550 (6)	0.02802 (12)	
Br2	0.25093 (7)	1.02533 (5)	0.34027 (6)	0.02714 (12)	
Hg1	0.38539 (2)	0.805335 (18)	0.216864 (18)	0.02106 (7)	
N1	0.1397 (5)	0.6727 (5)	0.2979 (4)	0.0223 (8)	
N2	−0.0327 (6)	0.3162 (5)	0.0956 (6)	0.0311 (10)	
N3	0.3219 (6)	0.8943 (5)	−0.0129 (5)	0.0238 (8)	
N4	0.4417 (7)	0.7012 (5)	−0.4646 (6)	0.0351 (11)	

### Atomic displacement parameters (Å^2^)

**Table:**

	*U*^11^	*U*^22^	*U*^33^	*U*^12^	*U*^13^	*U*^23^
C1	0.027 (2)	0.019 (2)	0.022 (2)	0.0020 (17)	−0.0076 (19)	−0.0081 (17)
C2	0.020 (2)	0.025 (2)	0.024 (2)	0.0023 (17)	−0.0048 (18)	−0.0038 (18)
C3	0.027 (2)	0.022 (2)	0.021 (2)	−0.0032 (18)	−0.0052 (19)	0.0010 (18)
C4	0.025 (2)	0.022 (2)	0.021 (2)	−0.0013 (18)	−0.0076 (18)	−0.0055 (17)
C5	0.024 (2)	0.024 (2)	0.025 (2)	−0.0017 (18)	−0.0073 (19)	−0.0041 (18)
C6	0.025 (2)	0.022 (2)	0.020 (2)	−0.0005 (17)	−0.0043 (18)	−0.0075 (17)
C7	0.035 (3)	0.024 (2)	0.025 (2)	0.002 (2)	−0.010 (2)	−0.0065 (19)
C8	0.055 (4)	0.025 (2)	0.026 (3)	0.010 (2)	−0.017 (3)	−0.007 (2)
C9	0.043 (3)	0.025 (2)	0.026 (2)	0.004 (2)	−0.016 (2)	−0.005 (2)
C10	0.025 (2)	0.021 (2)	0.020 (2)	−0.0017 (17)	−0.0062 (18)	−0.0037 (17)
C11	0.031 (3)	0.025 (2)	0.024 (2)	0.0001 (19)	−0.011 (2)	−0.0036 (19)
C12	0.027 (2)	0.021 (2)	0.024 (2)	−0.0018 (18)	−0.010 (2)	−0.0032 (18)
Br1	0.0268 (2)	0.0291 (2)	0.0289 (3)	0.00759 (19)	−0.0111 (2)	−0.01227 (19)
Br2	0.0334 (3)	0.0236 (2)	0.0239 (2)	0.00029 (19)	−0.0065 (2)	−0.01056 (18)
Hg1	0.02188 (10)	0.02100 (10)	0.02036 (10)	0.00031 (7)	−0.00584 (7)	−0.00698 (7)
N1	0.024 (2)	0.0245 (19)	0.0182 (18)	−0.0005 (15)	−0.0058 (16)	−0.0068 (15)
N2	0.030 (2)	0.029 (2)	0.034 (2)	−0.0031 (18)	−0.007 (2)	−0.0104 (19)
N3	0.023 (2)	0.0250 (19)	0.022 (2)	0.0014 (16)	−0.0068 (16)	−0.0052 (16)
N4	0.044 (3)	0.031 (2)	0.031 (2)	−0.001 (2)	−0.010 (2)	−0.009 (2)

### Geometric parameters (Å, °)

**Table:**

C1—N1	1.347 (6)	C7—H7	0.93
C1—C2	1.384 (7)	C8—C9	1.385 (8)
C1—H1	0.93	C8—H8	0.93
C2—C3	1.391 (7)	C9—C10	1.389 (7)
C2—H2	0.93	C9—H9	0.93
C3—C4	1.390 (7)	C10—C12	1.399 (7)
C3—H3	0.93	C10—C11	1.436 (7)
C4—C6	1.401 (7)	C11—N4	1.148 (7)
C4—C5	1.446 (7)	C12—N3	1.334 (7)
C5—N2	1.144 (7)	C12—H12	0.93
C6—N1	1.337 (6)	Br1—Hg1	2.4581 (5)
C6—H6	0.93	Br2—Hg1	2.4736 (5)
C7—N3	1.333 (7)	Hg1—N1	2.481 (4)
C7—C8	1.381 (8)	Hg1—N3	2.496 (4)
			
N1—C1—C2	122.7 (5)	C8—C9—C10	118.4 (5)
N1—C1—H1	118.7	C8—C9—H9	120.8
C2—C1—H1	118.7	C10—C9—H9	120.8
C1—C2—C3	120.0 (5)	C9—C10—C12	119.1 (5)
C1—C2—H2	120	C9—C10—C11	120.7 (5)
C3—C2—H2	120	C12—C10—C11	120.1 (4)
C4—C3—C2	117.1 (5)	N4—C11—C10	179.3 (6)
C4—C3—H3	121.4	N3—C12—C10	121.9 (5)
C2—C3—H3	121.4	N3—C12—H12	119
C3—C4—C6	120.0 (5)	C10—C12—H12	119
C3—C4—C5	121.2 (5)	Br1—Hg1—Br2	159.99 (2)
C6—C4—C5	118.8 (5)	Br1—Hg1—N1	98.01 (10)
N2—C5—C4	179.0 (6)	Br2—Hg1—N1	97.02 (10)
N1—C6—C4	122.1 (5)	Br1—Hg1—N3	97.22 (10)
N1—C6—H6	119	Br2—Hg1—N3	95.88 (10)
C4—C6—H6	119	N1—Hg1—N3	90.10 (14)
N3—C7—C8	123.4 (5)	C6—N1—C1	118.1 (4)
N3—C7—H7	118.3	C6—N1—Hg1	121.2 (3)
C8—C7—H7	118.3	C1—N1—Hg1	120.3 (3)
C7—C8—C9	118.7 (5)	C7—N3—C12	118.5 (5)
C7—C8—H8	120.6	C7—N3—Hg1	120.3 (3)
C9—C8—H8	120.6	C12—N3—Hg1	121.2 (3)
			
N1—C1—C2—C3	−0.3 (8)	C2—C1—N1—C6	0.0 (7)
C1—C2—C3—C4	−0.5 (7)	C2—C1—N1—Hg1	172.5 (4)
C2—C3—C4—C6	1.7 (7)	Br1—Hg1—N1—C6	−38.3 (4)
C2—C3—C4—C5	−179.7 (5)	Br2—Hg1—N1—C6	155.0 (4)
C3—C4—C5—N2	16E1(4)	N3—Hg1—N1—C6	59.0 (4)
C6—C4—C5—N2	−2E1(4)	Br1—Hg1—N1—C1	149.5 (4)
C3—C4—C6—N1	−2.1 (8)	Br2—Hg1—N1—C1	−17.2 (4)
C5—C4—C6—N1	179.3 (5)	N3—Hg1—N1—C1	−113.2 (4)
N3—C7—C8—C9	0.8 (10)	C8—C7—N3—C12	−0.2 (9)
C7—C8—C9—C10	−1.0 (10)	C8—C7—N3—Hg1	−178.2 (5)
C8—C9—C10—C12	0.7 (9)	C10—C12—N3—C7	−0.1 (8)
C8—C9—C10—C11	179.9 (6)	C10—C12—N3—Hg1	177.9 (4)
C9—C10—C11—N4	−6E1(5)	Br1—Hg1—N3—C7	−169.9 (4)
C12—C10—C11—N4	12E1(5)	Br2—Hg1—N3—C7	−5.0 (4)
C9—C10—C12—N3	−0.2 (8)	N1—Hg1—N3—C7	92.1 (4)
C11—C10—C12—N3	−179.3 (5)	Br1—Hg1—N3—C12	12.2 (4)
C4—C6—N1—C1	1.2 (7)	Br2—Hg1—N3—C12	177.1 (4)
C4—C6—N1—Hg1	−171.2 (4)	N1—Hg1—N3—C12	−85.9 (4)
